# Radiological findings in ancient Egyptian canopic jars: comparing three standard clinical imaging modalities (x-rays, CT and MRI)

**DOI:** 10.1186/s41747-018-0048-3

**Published:** 2018-06-20

**Authors:** Patrick E. Eppenberger, Mislav Cavka, Michael E. Habicht, Francesco M. Galassi, Frank Rühli

**Affiliations:** 1 Institute of Evolutionary Medicine, University of Zurich, Winterthurerstrasse 190, CH-8057 Zurich, Switzerland; 2 School of Medicine, University of Zagreb, Šalata 3, 10 000 Zagreb, Croatia; 3 School of Humanities and Creative Art (Department of Archaeology), Flinders University, Sturt Rd, Bedford Park, South Australia 5042 Australia

**Keywords:** Ancient Egyptian canopic jars, Computed tomography, Magnetic resonance imaging, Radiography, Paleoradiology

## Abstract

**Background:**

The aim of our study was to evaluate the potential and the limitations of standard clinical imaging modalities for the examination of ancient Egyptian canopic jars and the mummified visceral organs (putatively) contained within them.

**Methods:**

A series of four ancient Egyptian canopic jars was imaged comparing the three standard clinical imaging modalities: x-rays, computed tomography (CT) and magnetic resonance imaging (MRI). Additionally, imaging-data-based volumetric calculations were performed for quantitative assessment of the jar contents.

**Results:**

The image contrast of the x-ray images was limited by the thickness and high density of the calcite mineral constituting the examined jars. CT scans showed few artefacts and revealed hyperdense structures of organ-specific morphology, surrounded by a hypodense homogeneous material. The image quality of MRI scans was limited by the low amount of water present in the desiccated jar contents. Nevertheless, areas of pronounced signal intensity coincided well with hyperdense structures previously identified on CT scans. CT-based volumetric calculations revealed holding capacities of the jars of 626–1319 cm^3^ and content volumes of 206–1035 cm^3^.

**Conclusions:**

CT is the modality of choice for non-invasive examination of ancient Egyptian canopic jars. However, despite its limitations, x-ray imaging will often remain the only practicable method for on-site investigations. Overall, the presented radiological findings are more compatible with contained small organ fragments rather than entire mummified organs, as originally expected, with consequent implications for envisioned future sampling for chemical and genetic analysis.

## Key points

- Ancient Egyptian canopic jars were imaged using x-rays, CT and MRI.
- CT-based volumetric calculations revealed lower holding capacities of the jars than expected.
- CT is the modality of choice to image ancient Egyptian canopic jars.
- Portable x-ray systems remain the most practicable approach for on-site investigations.

## Background

### Paleoradiology and ancient Egyptian canopic jars

The use of medical imaging techniques, such as x-ray radiography, computed tomography (CT), x-ray micro-tomography (also called micro-CT) or magnetic resonance imaging (MRI), to study bioarchaeological materials can be summarised under the term ‘paleoradiology’. The era of paleoradiological research was initiated in 1894 by the physicist Walter Koenig (1859–1936) only a few months after Roentgen’s first publication of x-ray discovery [1]. Nowadays, even the use of CT already has a 40-year-old history in mummy research [2], generally describing pathologies, mummification techniques, artefacts and state of preservation [3–8]. Besides x-rays and CT, the feasibility of non-clinical MRI of bioarchaeological materials [9, 10] as well as other non-ionising imaging methods, such as terahertz imaging [11], has been demonstrated. CT has even been used to identify skeletal remains embedded in a soil matrix, in order to avoid destruction of the fragile specimens [4].

The composition of bioarchaeological specimens is often very heterogeneous, and materials of high density and atomic number, such as funerary accessories, jewelry, tools, weapons or body adornments, can be encountered [7, 12, 13]. On the other hand, bioarchaeological specimens may themselves be contained in dense vessels made of stone or ceramic. This is the case for ancient Egyptian canopic jars, which were used in the funerary setting in ancient Egypt between 2700 and 300 B.C. to separately store and preserve those internal organs that needed to be removed from the body in the course of the mummification procedure to avoid putrefaction, yet were considered essential for the afterlife [14–19].

Paleoradiology, therefore, often becomes a complex task, requiring unconventional methods and a good understanding of the characteristics of bioarchaeological materials and possible taphonomic and/or post-mortem alterations. In particular the loss of water in such samples, which leads to an increased density, makes it difficult to distinguish between different types of tissues or non-organic constituents. Substances employed during a mummification or embalming process, or later added during museum curation, can also alter tissue radiological appearance [20]. Furthermore, the use of imaging technologies for paleoradiology is often limited by additional factors, such as the availability and portability of the imaging equipment, financial costs and challenges obtaining permission to move or transport ancient specimens for examination.

### The aim of our study

This study was performed as part of a larger transdisciplinary mummy research project linking medicine, evolutionary biology and Egyptology [21–23]. The inventive focus of The Canopic Jar Project of the Institute of Evolutionary Medicine of the University of Zurich lies particularly on mummified human tissues contained in canopic jars and mummified visceral bundles, attempting to produce results otherwise not achievable by conventional ancient mummy research methods. The viscera (lung, liver, stomach, intestines) are particularly attractive targets for the investigation of pathogen evolution. Yet ancient Egyptian canopic jars have so far been widely neglected as objects for bioarchaeological research. In this setting, a basic non-invasive radiological examination performed before further invasive procedures, such as sampling for histological, chemical or genetic analysis, can provide valuable information on a canopic jar and its contents. This especially applies when examination of a larger number of canopic jars is planned, and one has to select which jars to sample. Furthermore, in the case of canopic jars with original sealing, the decision to be considered by museums’ curators on whether to open them or to keep them untouched may be assisted by preliminary radiological analysis as well.

To the best of our knowledge, there is no published data on comparative radiological investigation of ancient Egyptian canopic jars. The specific aim of our study was therefore to compare the potential and limitations of the three standard clinical imaging modalities, x-rays, CT and MRI, for the examination of canopic jars and the mummified visceral organs (putatively) contained within them. Additionally, our study aimed to qualitatively and quantitatively assess jar contents by CT-based density measurements and CT-based volumetric calculations.

## Methods

No institutional review board approval was necessary for this study. Nevertheless, this research project strictly committed to the code of ethics of the Institute of Evolutionary Medicine of the University of Zurich, which demands a careful judgment of the appropriateness of any research involving ancient human remains against the applied degree of invasiveness (http://www.iem.uzh.ch/en/institute/iemcodeofethics.html).

**Fig. 1:**
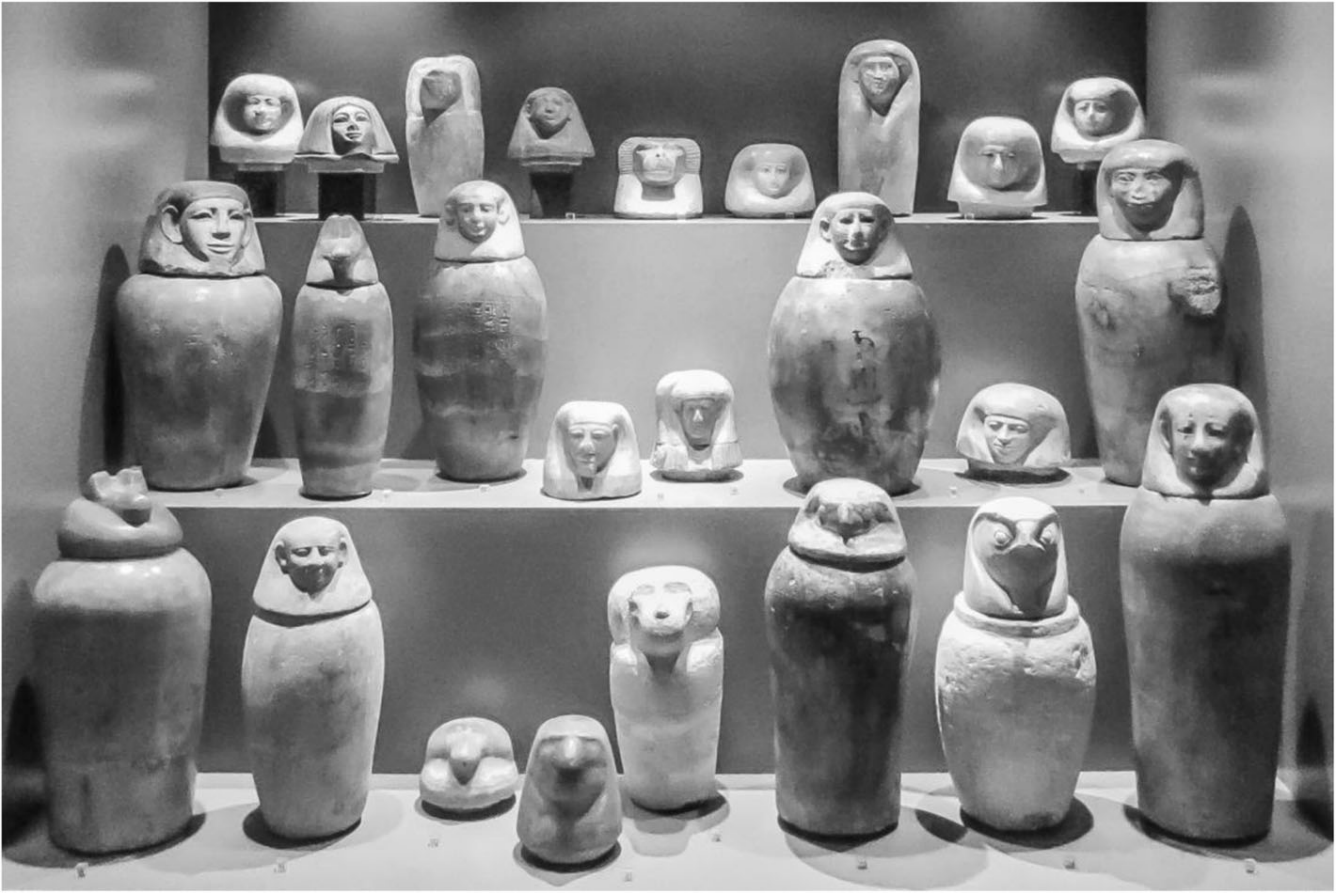
Ancient Egyptian canopic jars on display in the Egyptian collection of the Archaeological Museum in Zagreb, Croatia (with kind permission of the Archaeological Museum in Zagreb, Croatia)

Four canopic jars made of calcite, from the Egyptian collection of the Archaeological Museum in Zagreb, Croatia (inventory numbers 607, 610-2, 617 and 622-1), dated in the Late Period 26th–30th Dynasties (approximately 664–332 B.C.) [24], were subjected to the three standard clinical imaging modalities (x-rays, CT and MRI) at the University Department of Diagnostic and Interventional Radiology at the Dubrava University Hospital in Zagreb. See Fig. 1. Being part of the museum’s permanent Egyptian exhibition and because of their considerable insurance value, the canopic jars could only be taken out of the museum for one night.

CT scans were prioritised because of the planned volumetric calculations, followed by MRI and x-ray imaging. Art historical and Egyptological information regarding the inscriptions, lid figures and associated deities, which denote the putatively contained visceral organs (liver, lungs, stomach or intestines), was taken from the available museum catalogue [24].

### Computed tomography

CT scans were performed of all four canopic jars. The jars were placed in the scanner in axial orientation with the anterior side facing upward; the anterior side of the canopic being defined as the side of the inscription, which was recognisable on all four imaged jars. CT was performed on a multislice CT unit (Sensation 16, Siemens Healthcare, Erlangen, Germany). Acquisition parameters included a 16 × 0.75 mm collimation, a pitch of 0.45, a rotation time of 1.0 s, a tube voltage of 130 kVp and a tube current of 250 mA. Reconstruction parameters included a J30S medium smooth kernel for filtered back-projection, a 1-mm section thickness with a 0.7-mm increment and a field of view appropriate to head size.

### Magnetic resonance imaging

MRI scans of the three canopic jars with solid contents (inventory numbers 607, 617 and 622-1) were performed with positioning in analogy to the CT scans. MRI was performed on a 1.5-T scanner (Magnetom Avanto, Siemens Healthcare, Erlangen, Germany) equipped with gradient systems with a maximum strength of 33 mT/m and a maximum slew rate of 125 mT/m/ms. Based on our experiences with previous MRI scans of desiccated materials [9, 10], we used three-dimensional (3D) spoiled gradient-echo sequences with ultra-short echo times with the manufacturer’s array coils for the head and the spine (time of echo 0.07 ms, time of repetition 15 ms, flip angle 45°). A total of 40,000 radial projections were used for the reconstruction of 256 slices with 1.3 × 1.3 × 1.3 mm^3^ isotropic resolution, which resulted in an imaging time of approximately 10 min per jar. One canopic jar, which only contained small amounts of powdery material (inventory number 610-2), was not scanned for reasons of cost and time efficiency, since the observed displacement of the powder, when moving the jar between imaging systems, would not allow later correlation to CT.

### X-rays

X-ray images were taken in anterior-posterior projection using a clinical standard digital x-ray system (RADspeed Safire, Shimadzu Europa GmbH, Duisburg, Germany) equipped with a 65-kW x-ray generator, the anterior side of a canopic jar again being defined as the side where the inscription was located. Taking into account the high density and substantial thickness of the calcite mineral constituting the examined jars, a tube voltage of 150 keV, the highest possible voltage value on this system, and an exposure of 400 mA, the highest possible current value combined with 150 keV on this system, were used.

### Data storage, post-processing and measurements

**Fig. 2:**
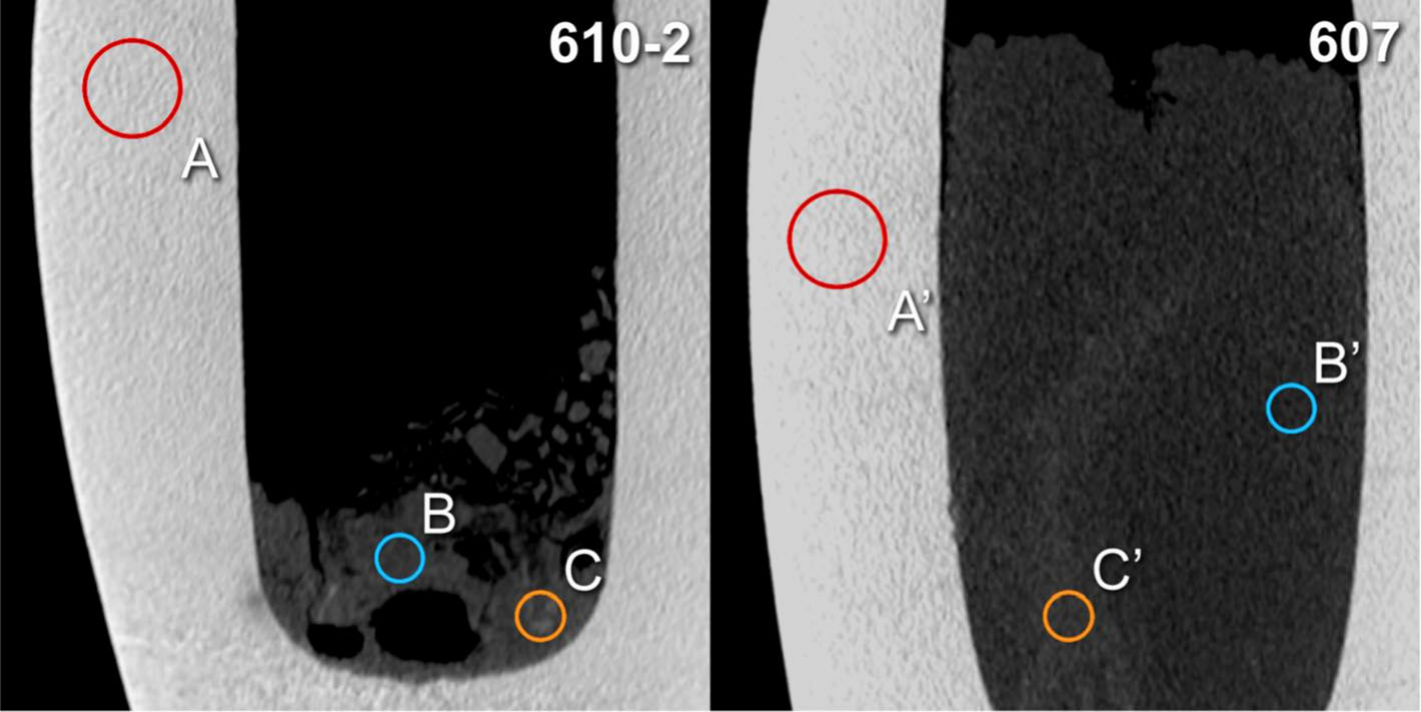
CT-based density measurements. Placement of regions of interest A and A’ (calcite mineral), B and B′ (surrounding material) and C and C′ (structures of higher radiodensity) for two canopic jars (inventory numbers 610-2 and 607) on sagittal multiplanar reconstructions (slab thickness 1.5 mm)

**Fig. 3:**
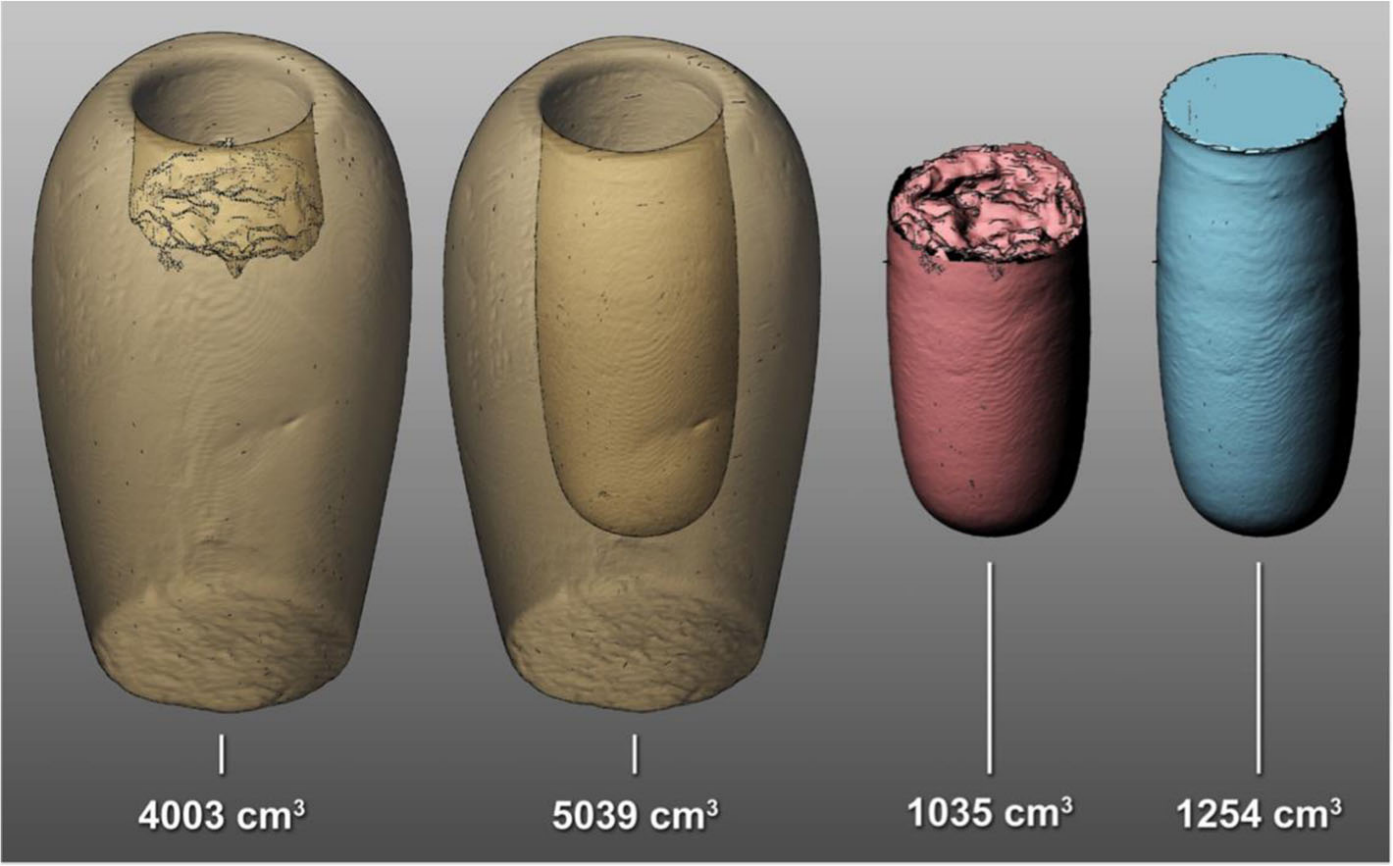
Three-dimensional surface reconstructions and volume calculations of one canopic jar (inventory number 607) obtained with Rhinoceros 3D. *From left to right*: volume including jar contents (−350 HU threshold), volume of calcite jar only (900 HU threshold), calculated volume of actual contents and calculated maximal holding capacity

All imaging data was automatically stored in the hospital’s picture archiving and communication system using a 12-bit grayscale Digital Imaging and Communications in Medicine format. The imaging data was later exported for viewing, post-processing and 3D volumetric reconstruction to a workstation (iMac 27” Retina 5K, 3.2GHz Quad Core i5, AMD Radeon R9 M390 2GB, 8GB RAM, 1 TB SSD; Apple Inc., Cupertino, CA, USA) running dedicated software (OsiriX MD, Pixmeo SARL, Bernex, Switzerland; Rhinoceros 3D, Robert McNeel and Associates, Seattle, WA, USA). Post-processing, including maximum intension projection, multiplanar reconstruction, 3D volume rendering and surface rendering using OsiriX as well as later volume calculation using Rhinoceros 3D, was performed by one of the authors. Density measurements in Hounsfield units (HU) were all conducted on sagittal cross sections through the centre of the jars (sagittal multiplanar reconstruction, maximal intensity projection, slab thickness 1.5 mm). For each jar, three circular regions of interest were placed in corresponding areas (calcite mineral, low-density material and identifiable structures of higher density), avoiding partial volume effects [18], as shown in Fig. 2. We also generated 3D surface reconstructions for later volumetric calculation in OsiriX using an automated thresholding method. Based on previously performed density measurements, 3D surface reconstruction was obtained twice for each analysed jar using two different density thresholds (−350 HU and 900 HU). This approach resulted in one 3D surface reconstruction (−350 HU) including the organic content and one 3D surface reconstruction (900 HU) excluding the organic content, comprising only the calcite mineral, for each analysed jar. The obtained polygonal 3D surface reconstructions were then exported in the stereo lithography file format in order to be imported into Rhinoceros 3D, where all dimensions and volumetric data could be directly calculated (Fig. 3) without further conversion of the polygonal 3D surface models.

### Assessment of image quality

Qualitative grading of all images was independently performed by two readers, a board-certified radiologist with more than ten years of experience in the field of paleoradiology and a paleoradiology researcher with eight years of experience in the field of diagnostic and investigational imaging research. Subjective image quality was assessed in terms of noise, differentiation among structures and overall diagnostic value. Noise was graded as follows: 4, very low; 3, low; 2, considerable with preserved diagnostic image quality; and 1, high, causing non-diagnostic image quality. The other parameters were scored as follows: 4, excellent; 3, good; 2, suboptimal, but still diagnostic; and 1, unacceptable (non-diagnostic). Grades for image quality were averaged across both readers as well as across modalities for further evaluation. No formal statistical analysis was performed, since this was only an experimental trial with four study objects to evaluate the feasibility and the potential of the three standard clinical imaging modalities (x-rays, CT and MRI).

## Results

Images of the canopic jars could be acquired with each of the three modalities (x-rays, CT and MRI) and post-processed as described above. Density measurements and volumetric calculations could be successfully derived from the CT scans. Results of the density measurements are listed in Table 1, and volumetric calculations are listed in Table 2; the results of our qualitative image grading are listed in Table 3.

**Table 1:** CT-based density measurements (HU)

Canopic jar inventory number	ROI A, calcite, diameter 20 mm Mean ± SD (range)	ROI B, surrounding material, diameter 10 mm Mean ± SD (range)	ROI C, putative tissue structures, diameter 10 mm Mean ± SD (range)
607	2158±176 (1607–2789)	186±90 (−88–448)	344 ± 92 (71–595)
610-2	2221±185 (1469–2823)	224±69 (38–455)	351 ± 150 (−16–790
617	2139±160 (1357–2653)	216±106 (−120–472)	373 ± 105 (−2–664)
622-1	2342±108 (2059–2763)	207±69 (−101–347)	309 ± 67 (−16–507)
Mean	2215±160 (1623–2757)	208±85 (−68–431)	344 ± 108 (9–639)

*ROI* region of interest, *SD* standard deviation

**Table 2:** CT-based volumetric calculations and dimensions

Canopic jar inventory number	Volume (cm^3^), holding capacity	Volume (cm^3^), alabaster only (threshold 900 UH)	Volume (cm^3^) including total contents (threshold −350 UH)	Volume (cm^3^), contents (calculated)
607	1254	4003	5039	1035
610-2	1020	3377	3673	295
617	1319	3680	4282	602
622-1	626	1681	1886	206
Canopic jar inventory number	Bore diameter (mm)	Bore depth (mm)	Total height of jar (mm)	Maximum diameter of jar (mm)
607	93	223	296	171
610-2	82	213	253	168
617	95	221	272	178
622-1	73	168	202	135

**Table 3:** Assessment of image quality

		Jar 607			Jar 617			Jar 622-1			Jar 610-2			Average		
		R1	R2	Average	R1	R2	Average	R1	R2	Average	R1	R2	Average	R1	R2	Average
CT	Noise and artefacts	4	3	3.50	4	4	4.00	3	4	3.50	3	4	3.50	3.50	3.75	3.63
	Differentiation of structures	3	3	3.00	3	2	2.50	3	2	2.50	3	2	2.50	3.00	2.25	2.63
	Overall diagnostic value	2	2	2.00	3	2	2.50	3	1	2.00	2	1	1.50	2.50	1.50	2.00
	Average	3.00	2.67	2.83	3.33	2.67	3.00	3.00	2.33	2.67	2.67	2.33	2.50	3.00	2.50	2.75
MRI	Noise and artefacts	2	2	2.00	2	2	2.00	2	2	2.00	Not performed			2.00	2.00	2.00
	Differentiation of structures	3	2	2.50	2	2	2.00	1	2	1.50				2.00	2.00	2.00
	Overall diagnostic value	2	1	1.50	2	1	1.50	1	1	1.00				1.67	1.00	1.33
	Average	2.33	1.67	2.00	2.00	1.67	1.83	1.33	1.67	1.50				1.89	1.67	1.78
X-rays	Noise and artefacts	Not performed			2	3	2.50	2	3	2.50	2	3	2.50	2.00	3.00	2.50
	Differentiation of structures				1	2	1.50	2	2	2.00	1	2	1.50	1.33	2.00	1.67
	Overall diagnostic value				1	1	1.00	1	1	1.00	1	1	1.00	1.00	1.00	1.00
	Average				1.33	2.00	1.67	1.67	2.00	1.83	1.33	2.00	1.67	1.44	2.00	1.72

*R1* reader 1, *R2* reader 2

Subjective image quality was assessed in terms of noise, differentiation of present structures and overall diagnostic value. Noise was graded as follows: 4, very low; 3, low; 2, considerable with preserved diagnostic image quality; 1, high, causing non-diagnostic image quality. The other parameters were scored as follows: 4, excellent; 3, good; 2, suboptimal, but still diagnostic; and 1, unacceptable and non-diagnostic

Overall, CT scans provided the best diagnostic image quality (average rating of 2.75), followed by MRI (1.78) and x-ray imaging (1.72).

### Computed tomography

**Fig. 4:**
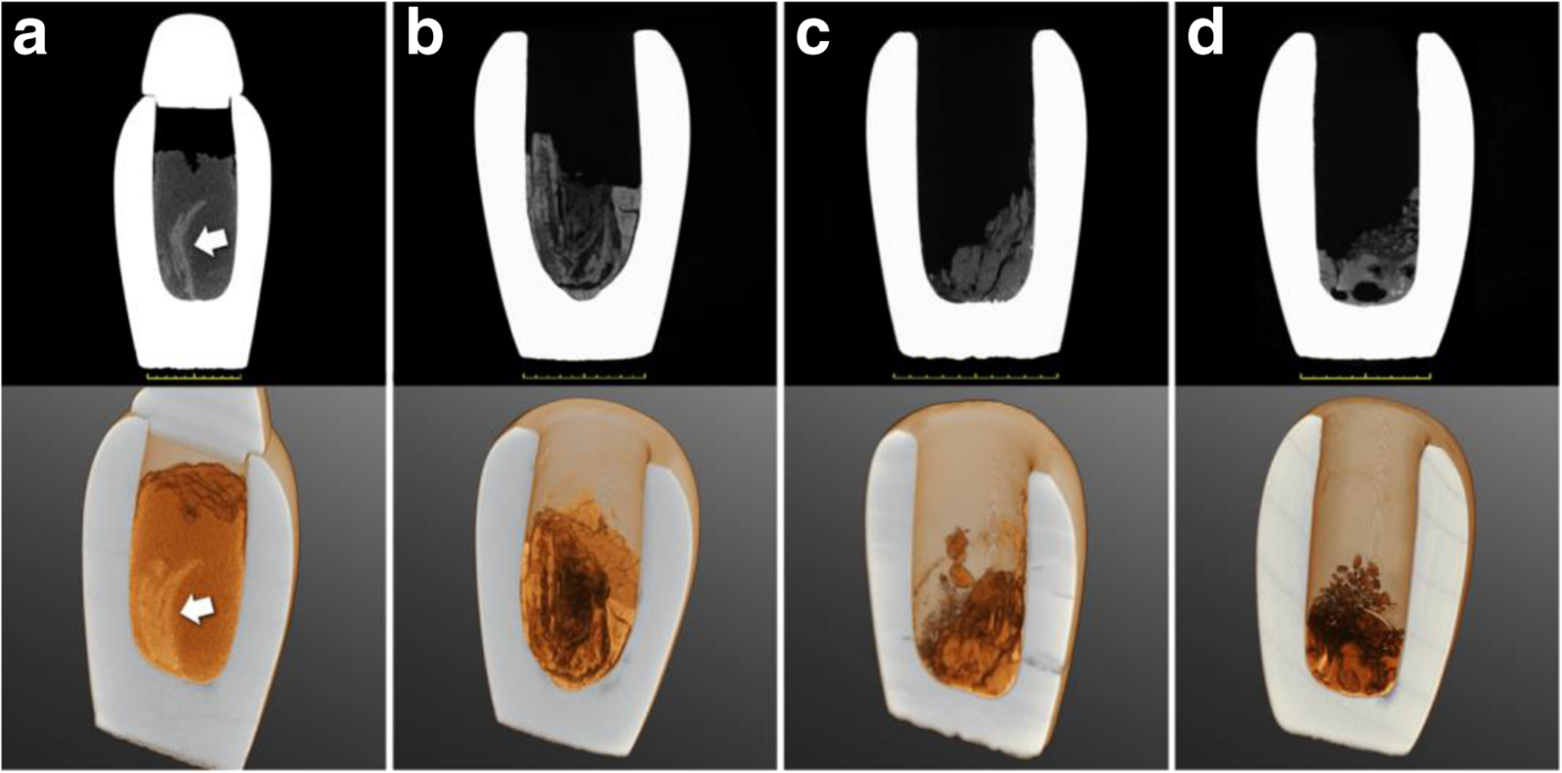
CT scans, sagittal cross sections through the centre of the jars (*top*) and correlative 3D volume renderings (*bottom*) of all four canopic jars (**a**, inventory number 607; **b**, inventory number 617; **c**, inventory number 622-1; **d**, inventory number 610-2). In panel **a**, *arrows* indicate a distinct hyperdense structure of organ-fragment-like morphology, probably intestine

CT scans revealed that all the jars are partially filled with material of mostly heterogeneous density (mean 208 HU, standard deviation [SD] 85 HU, range from −68 to 431 HU) (Fig. 4a–d). In one jar (inventory number 607), structures of distinct longitudinal morphology and higher density (mean 344 HU, SD 92 HU, range from 71 to 595 HU) were clearly distinguishable from a homogeneous surrounding material of lower density (mean 186 HU, SD 90 HU, range from −88 to 448 HU) (Fig. 4a). These structures, measuring approximately 10 cm in length and 3 cm in diameter, were located towards the centre of the bore and roughly oriented along the *z*-axis. Small areas of higher density surrounded by widely homogeneous material in the same density range were also identifiable in the other jars (Fig. 4b–d). The calcite mineral of which the jars are made proves to be of high radiodensity (mean 2215 HU, SD 160 HU, range from 1623 to 2757 HU). Nevertheless, CT scans showed only few artefacts, which were caused by hardening of the x-ray beam passing through several centimeters of calcite mineral [25]. Cupping artefacts were only relevant in the area of the central bore of the canopic jars, resulting in elevated densities in that area. Further artefacts, as known from metallic implants in clinical imaging, were absent.

Volumetric calculations revealed relatively low holding capacities for all jars, ranging from 626 to 1319 cm^3^, and actual content volumes between 206 and 1035 cm^3^, as listed in Table 2.

### Magnetic resonance imaging

**Fig. 5:**
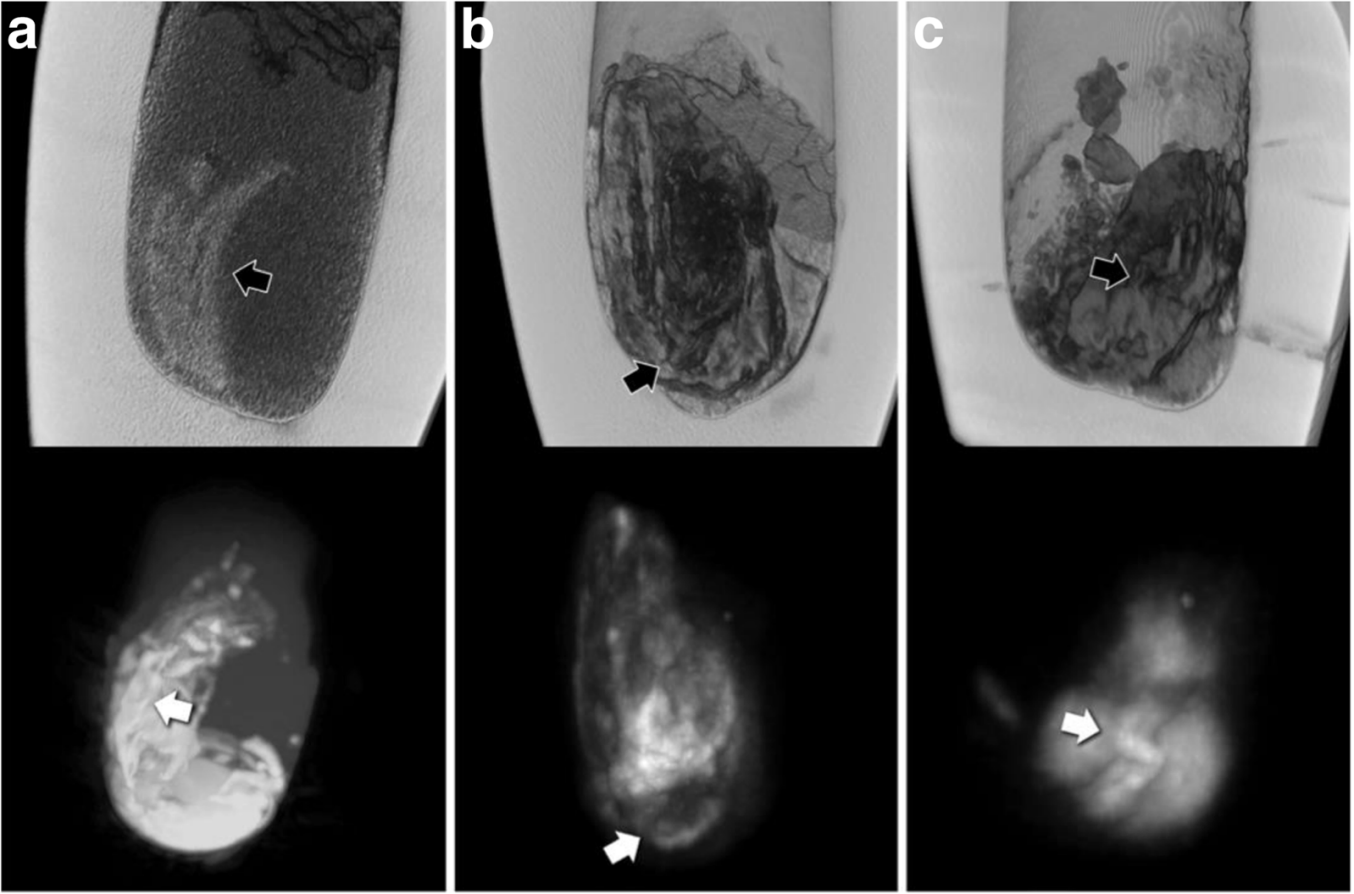
CT reconstructions (*top*) and correlative maximum intensity projections of MRI scans (*bottom*) of the three canopic jars with solid contents (**a**, inventory number 607; **b**, inventory number 617; **c**, inventory number 622-1). *Black arrows* (on CT) and *white arrows* (on MRI) indicate structures of organ-fragment-like morphology, probably intestine

MRI showed pronounced variations in signal intensity in the area of the central bore of the canopic jars, where the jar contents are located. No signal could, however, be detected from the surrounding calcite mineral of which the jars consist. In addition, the areas generating high signal intensity coincided well with structures of high density previously identified on the CT scans (Fig. 5a–c) in terms of shape, dimensions and orientation. In particular the outer border of these structures produced a very high signal. However, the signal intensity of the surrounding material seemed to coincide less with the findings identified on the CT scans.

### X-rays

**Fig. 6:**
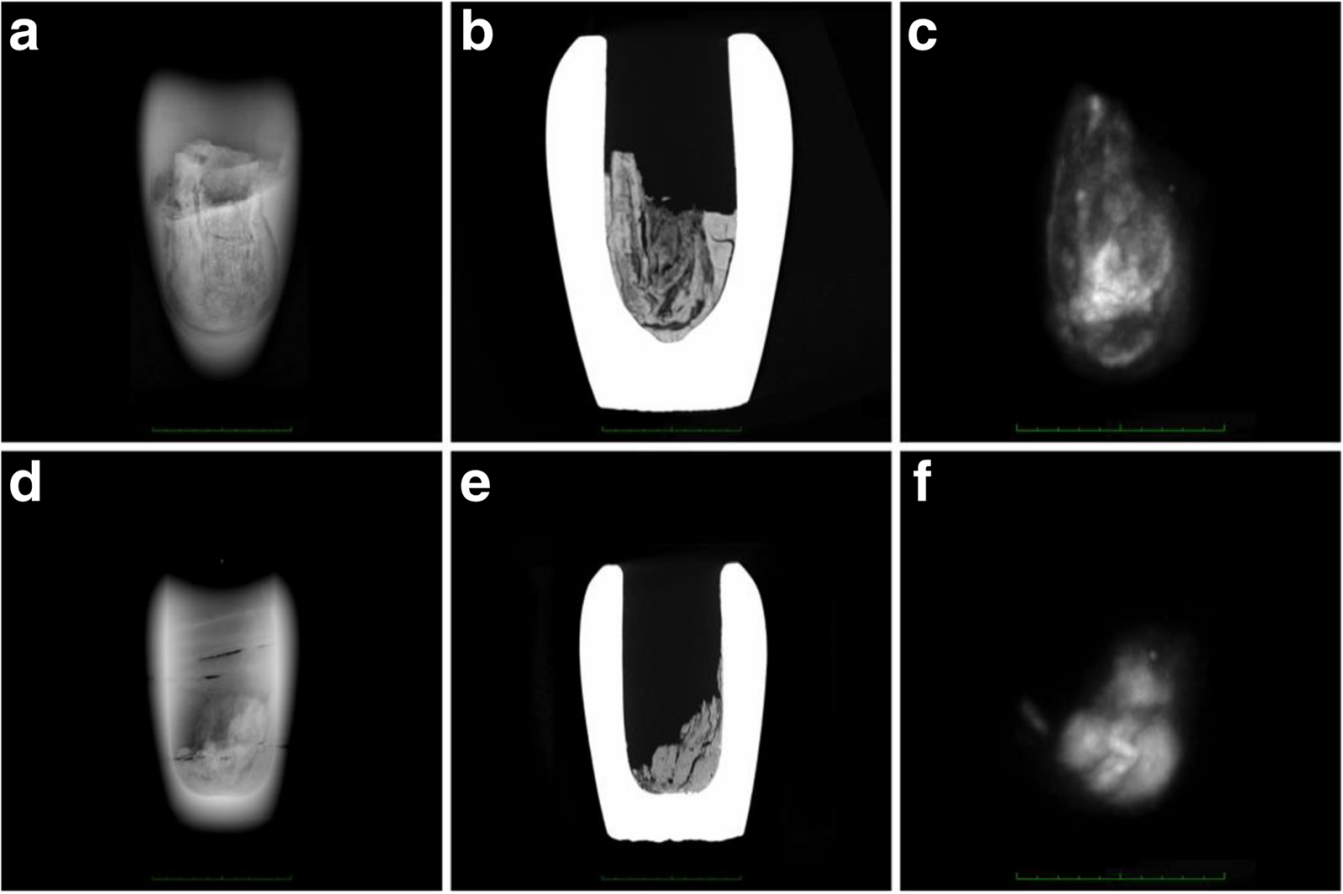
Direct comparison of the three imaging modalities: x-rays (**a**, **d**), CT (**b**, **e**) and MRI (**c**, **f**) of the canopic jars with the smallest and the largest diameter (**a**–**c**, inventory number 617; **d**–**f**, inventory number 622-1)

X-ray imaging provided limited image quality, mainly due to the high density and substantial thickness of the calcite mineral of which the examined canopic jars were composed. Nevertheless, inhomogeneous radiopaque material could be identified in the lower third of the three imaged canopic jars (Fig. 6a, d). In addition, in one jar (Fig. 6d), the superimposed calcite walls of the canopic jar showed great variations in density corresponding to cracks present in that jar’s calcite material.

## Discussion

Although x-ray imaging often remains the most practical imaging modality for many paleoradiological applications (since it can be performed on site, for example at a museum or in the field, using portable equipment), for the purpose of this study it was but of limited value. The poor image quality can be explained as a result of the substantial thickness and rather high density of the calcite mineral composing the examined canopic jars (mean 2215 HU, SD 160 HU), compared with the literature results for other minerals and non-metallic materials [7, 15]. Surprisingly, this did not much affect the CT scans, and — apart from slight beam hardening and cupping artefacts — no other masking effects could be found. Streaking artefacts were less of an issue due to the canopic jars’ cylindrical shape with a nearly symmetrical distribution of high-density areas along the *z*-axis.

However, the identifiable structures on one of the CT scans as well as the volumetric data on all the jars raise the question of whether the examined canopic jars do hold entire mummified human organs. In fact, due to the absence of primary textual evidence and because there are only a few sources of secondary literature by Herodotus (484–430 B.C.), Diodorus Siculus (first half of the first century B.C.), Strabo (approximately 63 B.C.–23 A.D.) and Pliny the Elder (23–79 A.D.), many aspects of ancient Egyptian mummification practice are not entirely understood.

The structures identified in the contents of canopic jar 607 would be far more compatible with a small organ fragment (potentially intestine) rather than an entire mummified organ. Strikingly, the corresponding MRI images can be viewed as highly compatible with the information provided by the CT scans. The observed pronounced variations in signal intensity coincide very well with the aforementioned structures of distinct morphology, identified with the CT scans, in particular for jar 607. Reasonably, it can be proposed that the image contrast observed in all three MRI scans was caused by organic mummification agents, which were absorbed by the mummified biological tissues. The fact that the outer border of these structures produced the most intense signal where most of the embalming substances would likely accumulate supports this interpretation. Another possible interpretation, however, could be that very thin gaps have formed between the dense structures identifiable on the CT scans and the surrounding material, which may have led to susceptibility artefacts on MRI.

As one would expect, CT in general provided far superior detail over MRI, essentially due to the higher spatial resolution, but further research will be necessary to determine the full capacity of MRI for this application. The fact that contrasts on MRI and CT complement each other for the examination of ancient Egyptian canopic jars is an advantageous result, encouraging further research.

It is also important to mention some of the limitations of this study. First, this study was of course limited by the small number of investigated canopic jars, which all stem from the Late Period (26th–30th Dynasties, from approximately 664 to 332 B.C.). Nevertheless, we are convinced that our results are in general representative for canopic jars made of calcite. Second, we did not image each of the four jars with all three modalities (x-rays, CT and MRI) due to the limited time during which we had both the canopic jars and the imaging equipment at our disposal. We prioritised CT scans for the intended volumetric calculations and selected only the smaller three jars for x-ray imaging. We also excluded one jar from MRI, as its contents were of powdery consistency, and displacement was inevitable when moving the jar between imaging systems, which would have hindered the correlation to CT scans. Compared to the standards applied in clinical radiological research, the level of diagnostic accuracy and the image quality of this study were mainly limited by the fact that the used imaging devices were conceived for clinical use with technical restrictions relevant for patient safety. This applies in particular for x-ray imaging, where a higher tube current would likely have improved the image quality. This study was nonetheless able to demonstrate the successful application of the three standard clinical imaging methods (x-rays, CT and MRI) on Egyptian canopic jars in particular and for similar objects containing human remains in general, thus contributing to broaden the technological spectrum for studies on historic human remains. In our opinion, the goal to achieve a higher degree of investigative output in studies on ancient human remains can be best achieved through a multimodal approach.

In conclusion, radiological techniques per se might not be considered indispensable, both for organ and pathology identification in ancient human remains. However, their implementation in the field of canopic jar research is important in the preliminary phase of studies which ultimately involve invasive testing. The presented radiological findings on the contents of the four examined ancient Egyptian canopic jars are, especially in the case of canopic jar 607, more consistent with small organ fragments rather than entire organs, as was hitherto assumed [18]. Radiological analysis of ancient Egyptian canopic jars by CT and MRI may therefore have made a significant contribution to a better understanding of ancient Egyptian mummification practice. However, even for canopic jars housed in European museums, the opportunity to perform examinations in a hospital will inherently remain limited. Therefore, portable x-ray imaging and, if possible, additional sampling will likely be the only practicable approach for most investigations of this kind.

## Abbreviations

3D: Three-dimensional
A.D.: Anno Domini
B.C.: Before Christ
CT: Computed tomography
HU: Hounsfield unit
MRI: Magnetic resonance imaging
SD: Standard deviation
